# Glutamate-induced obesity leads to decreased sperm reserves and acceleration of transit time in the epididymis of adult male rats

**DOI:** 10.1186/1477-7827-10-105

**Published:** 2012-12-05

**Authors:** Glaura SA Fernandes, Arielle C Arena, Kleber E Campos, Gustavo T Volpato, Janete A Anselmo-Franci, Débora C Damasceno, Wilma G Kempinas

**Affiliations:** 1 Biological Sciences Center, State University of Londrina, UEL, Londrina, PR, Brazil; 2 Department of Morphology, Institute of Biosciences, UNESP - UnivEstadualPaulista, Botucatu, SP, Brazil; 3 Institute of Biological and Health Sciences, Federal University of Mato Grosso, UFMT, Barra do Garça, MT, Brazil; 4 Department of Physiology, Ribeirão Preto Medical School, University of São Paulo, USP, Ribeirão Preto, SP, Brazil; 5 Laboratory of Experimental Research on Gynecology and Obstetrics, Department of Gynecology and Obstetrics, Botucatu Medical School, UNESP - UnivEstadualPaulista, Botucatu, SP, Brazil

**Keywords:** Obesity, Monosodium glutamate, Epididymis, Testosterone, Sperm, Rat

## Abstract

**Background:**

Given the established fact that obesity interferes with male reproductive functions, the present study aimed to evaluate sperm production in the testis and storage in the epididymis in a glutamate-induced model of obesity.

**Methods:**

Male rats were treated neonatally with monosodium glutamate (MSG) at doses of 4 mg/kg subcutaneously, or with saline solution (control group), on postnatal days 2, 4, 6, 8 and 10. On day 120, obesity was confirmed by the Lee index in all MSG-treated rats. After this, all animals from the two experimental groups were anesthetized and killed to evaluate body and reproductive organ weights, sperm parameters, plasma hormone levels (FSH, LH and testosterone), testicular and epididymal histo-morphometry and histopathology.

**Results:**

Significant reductions in absolute and relative weights of testis, epididymis, prostate and seminal vesicle were noted in MSG-treated animals. In these same animals plasma testosterone and follicle-stimulating hormone (FSH) concentrations were decreased, as well as sperm counts in the testis and epididymis and seminiferous epithelium height and tubular diameter. The sperm transit time was accelerated in obese rats. However, the number of Sertoli cells per seminiferous tubule and stereological findings on the epididymis were not markedly changed by obesity.

**Conclusions:**

Neonatal MSG-administered model of obesity lowers sperm production and leads to a reduction in sperm storage in the epididymis of adult male rats. The acceleration of sperm transit time can have implications for the sperm quality of these rats.

## Background

Obesity is increasing worldwide and it represents a challenge to the scientific community being major public health concern [1]. It is a heterogeneous disorder with wide variations in risks for complicating diseases [2] and has been associated with an increased risk of many serious illnesses such as cardiovascular diseases, diabetes mellitus and some types of cancer [3].

Although the negative effects of obesity on reproductive function were first documented more than 2000 years ago by Hippocrates [4], the etiology of the adverse effects of this relationship has not been studied in depth. There is little data on human male reproductive outcomes, but have shown that overweight and obese men present hormonal changes such as lower plasma levels of sex hormone-binding globulin (SHBG), total testosterone, free testosterone and follicle-stimulating hormone (FSH) [5-7]. Nevertheless, lower sperm counts [8,9], poor semen quality [7], decreased normal-motile sperm cells and increased DNA fragmentation index [10] were described in men that showed increased body mass index (BMI). Recent information obtained from different population studies suggests an inverse relationship between the increase in body mass index and fertility [7,11]. However, there were few studies that relate obesity to male reproductive dysfunction, and the existents are inconclusive. According to Sallmen et al. [9], programs to prevent obesity may improve men’s reproductive health and save medical costs for infertility treatment.

Due to the difficulty of studying obesity-induced reproductive complications in men, experimental models have been used. The induction of obesity may be performed in animals by neuroendocrine, dietary or genetic changes [12]. According to one of these models if neonatal rats are treated with monosodium glutamate (MSG) in the neonatal period, they become obese through the course of development [13-19]. Neonatal MSG treatment leads to the destruction of specific sites in the hypothalamus, including ventromedial and arcuate nuclei, in turn provoking disorders in the control of absorption and energy-expenditure-specific mechanisms resulting in a state of obesity [20,21].

No study evaluated the effects of this obesity model on the epididymal structure or function -organ responsible for sperm maturation and survival [22]. Thus, the present study aimed to evaluate the histopathology, morphometry and storage sperm in the epididymis in obese male rats due to neonatal MSG treatment. Nevertheless, plasma hormonal assay, sperm production and morphometric analysis in the testis were also evaluated.

## Methods

### Parental generation

Wistar rats (males, n = 4 and females, n = 10) weighing about 180 g (90 days of life), were adapted for seven days in the Laboratory of Experimental Research of Gynecology and Obstetrics, Department of Gynecology and Obstetrics. The rats were kept in collective cages in controlled conditions of temperature (22 ± 3°C), light (12h light/dark cycle) and relative humidity (60 ± 5%). The animals were fed laboratory chow (Purina®) and tap water *ad libitum*. All experimental procedures carried out in this study were approved by the Committee for Ethics in Animal Experimentation at the School of Medicine of Botucatu - Unesp, São Paulo State, Brazil(Protocol number: 318/2003).

Females (n = 10) were mated overnight with normal males and sperm presence in vaginal wet smears in the following morning was defined as day zero (0) of pregnancy. Pregnant rats were kept in individual cages during the pregnancy period (21 days), including vaginal delivery and lactation periods (21 days).

### Experimental rats

From the offspring obtained, eight newborns (NB) were kept with their dams (one newborn/nipple, preferentially males) throughout the lactation period (21 days). When the number of males would not reach eight, female NBs were placed in the litter. This procedure was utilized to obtain better and equal feeding for all NBs.

Male offspring were divided into two groups: rats that received saline solution (2.0% NaCl – Control) subcutaneously (sc) at postnatal days 2, 4, 6, 8 and 10 (Control group, n = 9) and rats given a solution of monosodium glutamate (MSG - 4.0 mg/g body weight, sc) at same days as those of the control group (MSG group, n = 9) [15,19,23-25]. After weaning, male rats were transferred to collective cages (4 animals per cage) and kept in controlled conditions. This methodology is related to induce lipohypertrophyin periepididymal fat [15].

### Obesity parameter of male rats

The parameter of obesity was obtained by Lee index for each male rat at month 4 of life (120 days). This index was defined as the cube root of body weight (g) x 10 / naso-anal length (cm), for which a value equal to or less than 0.300 was classified as normal. Rats presenting values higher than 0.300 were classified as obese [24,26] and included in this experiment.

### Body weight and weight of male reproductive organs

In adult age (120 days old), male rats from two experimental groups (n = 9; 1 per litter) were weighed and killed by decapitation. The left testis and epididymis, vas deferens, ventral prostate and seminal vesicle (without the coagulating gland and full of secretion) were removed and their absolute and relative weights determined.

### Plasma hormonal assay

After decapitation of each rat, blood (between 0900 and 1100 h am) from the ruptured cervical vessel was collected in a heparinized tube and evaluated for the determination of plasma testosterone (T), follicle-stimulating hormone(FSH) and luteinizing hormone (LH) levels. The plasma was obtained after centrifugation (2400 rpm, 20 min, 3.5°C) in a refrigerated apparatus and was frozen at – 20°C until the moment of hormonal determination. Plasma hormone levels were determined by double-antibody radioimmunoassay, using the Testosterone Maia® kit (BiochemImmuno System) and specific kits for FSH and LH, at the Neroendocrinology Laboratory, Dental School of Ribeirão Preto, University of São Paulo - USP. All the samples were dosed in the same assay, to avoid inter-assay errors. The lower detection limit was 0.064 ng/mL, with a 4% intra-assay error for T; 0.04 ng/mL, with a 3.4% intra-assay error for LH; and 0.09ng/mL, with a 2.8% intra-assay error for FSH.

### Daily sperm production per testis, sperm number and transit time in the epididymis

Homogenization-resistant testicular spermatids (step 19 of spermiogenesis) and sperm in the caput/corpus epididymis and cauda epididymis were enumerated as described previously by Robb et al. [27], with adaptations adopted by Fernandes et al. [28]. Briefly, each left testis, decapsulated and weighed soon after collection, was homogenized in 5 ml of NaCl 0.9% containing Triton X 100 0.5%, followed by sonication for 30 sec. After a 10-fold dilution a sample was transferred to Newbauer chambers (4 fields per animal), preceding a count of mature spermatids. To calculate daily sperm production (DSP) the number of spermatids at step 19 was divided by 6.1, which is the number of days of the seminiferous cycle in which these spermatids are present in the seminiferous epithelium. Then, the DSP per gram was calculated in order to determine the efficiency of the process [29]. In the same manner, caput/corpus and cauda epididymidis portions were cut into small fragments with scissors and homogenized, and sperm counted as described for the testis. The sperm transit time through the epididymis was determined by dividing the number of sperm in each portion by the DSP.

### Histopathological evaluation

The right testis and epididymis in 5 animals per group were removed and fixed in Alfac fixing solution (80% ethanol, formaldehyde and glacial acetic acid, 8.5:1.0:0.5, v/v) for 24 h. The pieces were embedded in paraffin wax and sectioned at 7 μm. The sections were stained with hematoxylin and eosin (H&E).

### Morphometric and stereological analysis

Using an imaging analysis system (Leica Q-Win Software version 3 for Windows™), the testicular morphometry and stereological analyses of the epididymis (cauda, region 6A) were accomplished by utilizing H&E sections [28]. Morphometric analysis was performed to evaluate seminiferous epithelium height and tubular diameter. For this study 10 sections per animal were measured (stage IX of spermatogenic cycle) at X 200 magnification.

Random H&E images of 75 histological fields per experimental group were captured and analyzed by the stereological method such that histological fragments of all animals were evaluated equally (15 per animal). Stereological analyses were obtained by Weibel’s multipurpose graticulate, with 120 points and 60 test lines [30] to compare the epididymal components (epithelium, stroma and lumen) in the experimental groups.

### Number of Sertoli cells per seminiferous tubule

To evaluate the process of proliferation of Sertoli cells, nuclei were counted in histological cuts (5-μm-thick) from the testis of rats at adulthood, in 20 seminiferous tubules per rat at stage VII of spermatogenesis, classified according to Leblond and Clermont [31].

### Statistical analysis

For comparison of results between the experimental groups, the Student’s *t*-test or Mann–Whitney test was employed according to the characteristics of each variable. The results were considered significant for *p<0.05*.

## Results

Table 1 shows the Lee index range found in both groups. Although there was data for obesity in control group in some rats, obesity was totally confirmed by Lee index in all rats of MSG - treated group (Table 1) which showed significant diminished body weight and naso-anal length in relation to control group. Nevertheless, male obese rats presented significant reductions in absolute and relative weights of the testis, epididymis, prostate and seminal vesicle (Table 1). Plasma testosterone and FSH levels were significantly diminished in male obese rats. However, plasma LH levels presented similarity between groups (Table 2).

**Table 1:** Lee index range, naso-anal length,final body weight and absolute and relative weights of male reproductive organs from control and MSG groups

**Groups**	**Control (n = 9)**	**MSG (n = 9)**
Lee index range	0.189 – 0.314	0.369 – 0.424
Final body weight (g)	487.30 ± 12.10	421.40 ± 10.72*
Naso-anal length (cm)	24.10 ± 0.25	18.50 ± 0.50*
Testis (g)	1.66 ± 0.08	1.00 ± 0.06*
Testis (g/100g)	0.34 ± 0.02	0.24 ± 0.01*
Epididymis (mg)	668.83 ± 20.00	392.00 ± 32.24*
Epididymis (mg/100g)	138.42 ± 5.17	93.00 ± 6.60*
Ventral prostate (mg)	605.50 ± 38.74	220.00 ± 68.70*
Ventral prostate (mg/100g)	125.11 ± 9.00	50.96 ± 15.70*
Seminal vesicle (g)	1.70 ± 0.18	0.44 ± 0.10*
Seminal vesicle (g/100g)	0.35 ± 0.03	0.10 ± 0.02*

Values are expressed as mean ± SEM. * p < 0.05 (Student’s *t*-test).

**Table 2:** Plasma hormonal levels from control and MSG groups

**Groups**	**Control (n = 9)**	**MSG (n = 9)**
FSH (ng/mL)	7.05 ± 0.79	4.86 ± 0.64*
LH (ng/mL)	3.05 ± 0.87	3.83 ± 0.90
Testosterone (ng/mL)	1.52 ± 0.19	0.57 ± 0.06*

Values are expressed as mean ± SEM. *p < 0.05 (Student’s *t*-test).

Experimental obesity led to a significant decrease in the numbers of testicular spermatids and sperm in the caput/corpus and cauda epididymidis, and a significant decrease in daily sperm production (DSP) (Table 3). However, there was no significant decrease in DSP/g of testis in the MSG group. In the male obese rats there was also a significant reduction in the sperm transit time in the cauda epididymidis, but not in the caput/corpus epididymidis (Table 3).

**Table 3:** Epididymal and testicular sperm parameters from control and MSG groups

**Groups**	**Control (n = 9)**	**MSG (n = 9)**
Daily sperm production (x 10^6^/testis/day)	43.13 ± 1.62	21.90 ± 1.24*
Daily sperm production (x 10^6^/g of testis/day)	27.30 ± 0.81	26.87 ± 1.81
Sperm count in the testis (x 10^6^/organ)	263.00 ± 10.00	134.70 ± 8.00*
Relative sperm count in the testis (x 10^6^/g of organ)	166.50 ± 5.00	163.83 ± 11.10
Sperm count in the caput/corpus epididymis (x 10^6^/organ)	172.15 ± 5.60	76.43 ± 5.60*
Relative sperm count in the caput/corpus epididymis (x 10^6^/g of organ)	516.01 ± 20.24	382.56 ± 18.00*
Sperm count in the cauda epididymis (x 10^6^/g)	335.16 ± 11.85	126.13 ± 22.12*
Relative sperm count in the cauda epididymis (x 10^6^/g of organ)	1122.00 ± 27.27	861.16 ± 42.34*
Sperm transit time in the caput/corpus (days)	4.00 ± 0.09	3.51 ± 0.23
Sperm transit time in the cauda (days)	7.90 ± 0.46	5.70 ± 0.84*

Values are expressed as mean ± SEM. * p < 0.05 (Student’s *t*-test).

Histopathological evaluation of the testis and epididymis did not reveal treatment-related morphological alterations (data not shown). The morphometric testicular analysis showed significant reductions in tubular diameter and seminiferous epithelium height in male obese rats (Table 4). The Sertoli cell number per seminiferous tubule (Control: 18.46 ± 1.57 and MSG: 16.02 ± 1.83; p > 0.05 - mean ± S.E.M.) and the epididymal stereological analysis showed similarity between the groups (Table 4).

**Table 4:** Morphometric and stereological analyses from control and MSG groups

**Groups**	**Control (n = 5)**	**MSG (n = 5)**
**Testicular morphometry**		
Tubular diameter	289.82 ± 4.14	269.61 ± 3.72*
Epithelium height	80.02 ± 1.40	75.86 ± 1.40*
^**1**^**Epididymal stereology**		
Epithelium	5.95 [4.76 – 7.74]	6.55 [4.76 – 7.74]
Stroma	25.60 [16.81 – 26.04]	21.43 [19.05 – 31.70]
Lumen	68.45 [67.26 – 77.38]	70.83 [62.36 – 75.60]

Values are expressed as mean ± SEM. *p < 0.05 (Student’s *t*-test).

^1^Values are expressed as median followed by quartile intervals [Q1-Q3]. *p* > 0.05 (Mann Whitney test).

## Discussion

Our study demonstrates that neonatal treatment with MSG alters the epididymal parameters by reducing sperm storage and accelerating sperm transit time. Furthermore, the present data confirm previous results showing that neonatal treatment with MSG is able to induce obesity (high Lee index, small corporal weight and naso-anal length) [32-36] although few control rats also presented obesity status by Lee index, related to aging [19]. Thus, neonatal treatment with MSG caused a cessation of growth and development with a concomitant accumulation of body fat leading to a decrease in body weight in relation to control group. Neonatal MSG treatment is a model of obesity in rodents which causes alterations in hypothalamic arcuate nucleus (ARC) and impairs leptin and insulin signaling in this region [37-39] resulting in hyperleptinemia and hyperinsulinemia. When the hypothalamic ventromedial nucleus and arcuate nucleus are destroyed in rats by treatment with MSG in the neonatal stage, obesity occurs as the rats grow [40].

Wet-weight alterations in the reproductive organs constitute a parameter for indicating changes in sex hormone levels [41]. In this study, the significant reduction in the weights of all analyzed organs in obese rats corroborate with the reduction of testosterone levels. As mentioned previously, the obesity induced by glutamate is obtained through a hypothalamic injury that disrupts the secretion of hormones including the gonadotrophins (FSH and LH) [42,43]. According to França et al. [18], in this obesity model, significant diminutions in the testosterone and FSH levels are related to disrupted HPG axis development due to the hyperleptinemia. In the same manner, Tena-Sempere et al. [44] reported that a possible cause for this severe reduction in the testosterone levels in animals neonatally treated with glutamate may have been due to an increase in plasma leptin levels. However, despite the decreased testosterone and FSH levels, the LH levels (hormone directly involved in regulating testosterone secretion) were unchanged by MSG treatment. This result can be related with severe ARC lesions [24,45,46] and so we suggest that MSG specifically leads to lesions in FSH neurons. Moreover, it is important to emphasize that the hormonal profile of obese men is characterized by decreased total and, often, free testosterone levels, diminished gonadotropin levels, and increased circulating estrogen levels [47].

The number of spermatids present in the testis and the total DSP are important indicators of male fertility potential [29]. In the present study, the reduction in sperm production led by MSG-induced obesity was associated with reduced testicular weight, seminiferous tubular diameter, testicular seminiferous epithelium height and testosterone and FSH levels. On the other hand, França et al. [18] showed unchanged sperm production, testis weight and seminiferous tubular diameter in obese adult rats after neonatal glutamate-induced obesity. In this sense, it is known that the number of Sertoli cells, established during the prepubertal period, determines the final testicular size and the magnitude of sperm production in sexually mature animals [48-50]. In the present study the number of Sertoli cells did not change in the obese rats, as well as the efficiency of the spermatogenic process, as evidenced by results of DSP per gram of testis that were similar between the experimental groups. It is possible that the disturbance in the HPG axis due to MSG treatment compromises the real significance of reduced FSH plasma levels observed in the present model. Evaluation of inhibin B levels in MSG rats could provide additional information regarding Sertoli cell function [18]. Thus, this experimental model may produces lightly different results between different research groups, as disclosed in the literature.

The reduction in sperm count in the epididymis in obese rats was probably responsible by the decrease of the epididymal weight and contributed for the acceleration of the sperm transit time through the epididymis. Acceleration in sperm transit time can impair sperm maturation and reduce the number of gametes available for ejaculation and fertility [51]. These alteration were independent of epididymal components (epithelium, stroma and lumen) since they are unaffected by glutamate-induced obesity.

It is very known that in obesity conditions there is an excess of leptin which is secreted by the adipocytes [44]. Studies show that the plasma leptin concentrations were significantly elevated in both humans [52,53] and in rodent obesity models [54,55], and directly proportional to the amount of fat. Leptin is important for the regulation of food intake, energy metabolism and reproductive function [55-58]. It has been proposed that a specific narrow leptin concentration range is necessary to maintain normal reproductive function, and levels below or above these thresholds are critical to this peptide’s influence on the function of the hypothalamus-pituitary-gonadal (HPG) axis [57,58] by acting on hypothalamic receptors and also by promoting peripheral effects [17]. However, the relationship between high leptin levels and the male reproductive system is not clear.

## Conclusions

In conclusion, the neonatal MSG-administered model of obesity lowers sperm production and leads to a reduction in sperm storage in adult male rats. Moreover, the acceleration of sperm transit time can have implications for the sperm quality of these rats. Thus, further studies should address (sperm motility, DNA integrity and fertility assay) the relationship between glutamate-induced obesity and epididymis function.

## Competing interests

The authors declare that they have no competing interests.

## Authors’ contributions

All authors participated in the design, interpretation of the studies, analysis of the data and review of the manuscript; GSAF and ACA conducted the experiments; KEC, GTV and DCD induced the obesity; JAAF performed hormone assay; and GSAF, ACA and WDGK performed data analyses and wrote the manuscript. All authors read and approved the final manuscript.
